# Synthesis and Characterization of the Zinc-Oxide: Tin-Oxide Nanoparticle Composite and Assessment of Its Antibacterial Activity: An In Vitro Study

**DOI:** 10.7759/cureus.53016

**Published:** 2024-01-26

**Authors:** Arshya A Kumar, Ravindra Kumar Jain

**Affiliations:** 1 Department of Orthodontics and Orthopedics, Saveetha Dental College and Hospitals, Saveetha Institute of Medical and Technical Sciences, Saveetha University, Chennai, IND; 2 Department of Dentistry, Saveetha Dental College and Hospitals, Saveetha Institute of Medical and Technical Sciences, Saveetha University, Chennai, IND

**Keywords:** dental, biomedical, sol-gel technique, nanoparticles, tin oxide, zinc oxide

## Abstract

Introduction

Nanoparticles (NPs) have been widely used for biomedical applications. Various methods of synthesis of NPs have been performed and the sol-gel technique is one of the most common and feasible methods. ZnO and SnO_2_ NPs are widely used due to their interesting properties and versatile medical applications. The present study aimed to synthesize a composite of ZnO- SnO_2_ NPs and evaluate its structural, morphological, and antibacterial properties.

Materials and methods

ZnO-SnO_2_ NPs were prepared via the sol-gel technique. The morphological study was performed by scanning electron microscopy (SEM) imaging, the structural study was performed by X-ray diffraction (XRD) analysis, and chemical studies were performed by Fourier transform infrared spectroscopy (FT-IR) and energy-dispersive X-ray spectroscopy (EDAX). Antibacterial properties of the NPs were assessed by the agar diffusion test and the area of bacterial growth that was inhibited was measured under high and low concentrations of the NPs.

Results

The SEM analysis confirmed the irregular shape and elemental composition of the synthesized NPs. The purity of the NPs was confirmed by the EDAX spectrum, which indicates the weight percentages of the elements in the NPs as follows: Sn-53.8%, Zn-12.5%, O-29.1%, and C-4.7%. The chemical bonds between the NPs were confirmed by Fourier transform infrared spectroscopy. XRD analysis confirmed the high degree of crystallinity of the NPs and orthorhombic structure of SnO_2 _and the hexagonal structure of ZnO. The zone of inhibition against S. *aureus*, S. *mutans,* and E. *coli* for low concentrations of the NPs was 24 mm, 26 mm, and 30 mm and for high concentrations of the NPs it was 26 mm, 28 mm, and 31mm and these values were similar to the control antibiotics.

Conclusion

ZnO- SnO_2_ NPs were successfully prepared by the sol-gel method. The presence of NPs was confirmed and successfully characterized. The prepared NPs had a good antimicrobial effect against the tested pathogens.

## Introduction

The use of nanotechnology has unleashed unlimited potential and it has been used in both industrial and biomedical fields. These nanostructures have a high surface-to-volume ratio, thereby increasing the free surface energy and ability to alter their chemical and physical properties, thus increasing their reactivity manifold [1-3]. The production of nanoparticles (NPs) can be accomplished by chemical and mechanochemical methods and the most commonly used processes for the synthesis of NPs include gas condensation, vacuum deposition and evaporation, chemical vapor deposition and condensation, mechanical attrition, chemical precipitation, sol-gel, and electrodeposition [4].

A zinc oxide (ZnO) NP is an odorless white powder widely used for its optical, electrical, photochemical, and catalytic properties [1,5]. Previous studies have reported anti-inflammatory, antifungal, antibacterial, antidiabetic, anticancer, wound healing, bioimaging, and drug carrier properties of ZnO NPs [6-10]. When coated on orthodontic brackets and wires, they are known to minimize surface roughness, hence decreasing friction and overall treatment time [11,12]. When included in resin composites, they have demonstrated excellent physical and mechanical qualities [13-15]. Tin has been coated on orthodontic stainless arch wires and improved tensile strength, load-bending strength, and reduced frictional resistance have been noted. Tin oxide NPs have biomedical applications and are reported to have photocatalytic, antioxidant, and antimicrobial properties [16].

ZnO/SnO_2_ nanocomposites were successfully prepared using the sol-gel method and then characterized by Kumar et al. [17]. The sol-gel method of synthesis of NPs involves hydrolysis and polymerization reactions, followed by heating the gel and vaporizing the solvent to obtain the final product. This simple method of NP synthesis provides a homogeneous powder of NPs and is feasible; hence, it is more popular when compared to other methods [18,19]. No previous studies have reported on the preparation of ZnO/SnO_2_ nanocomposites by the sol-gel method, followed by testing their effect on common oral pathogens. The present study aimed to synthesize a composite of ZnO-SnO_2_ NPs and evaluate their morphological, structural properties, and antibacterial properties at two different concentrations.

## Materials and methods

Synthesis of zinc oxide-tin oxide NPs

The sol-gel method of synthesizing NPs was employed to produce ZnO-SnO_2_ NPs. To create a homogeneous solution, 0.4 M tin chloride pentahydrate (SnCl_2_.5H_2_O) was first dissolved in double-deionized water. Next, 8 M sodium hydroxide (NaOH) was added dropwise at a steady rate. The SnCl_2_.5H_2_O solution was mixed with the ZnO precursor zinc sulfate heptahydrate (ZnSO_4_.5H_2_O) at an optimized concentration of 1 M. The complete solution was continuously stirred until a homogenous solution was obtained. The solution was then agitated for 20 minutes before being microwaved at 320 W for 10 minutes. To get rid of the contaminants and impurities, the resultant product was centrifuged and cleaned using deionized water and ethanol alternately five times. After drying and annealing at 500 °C for 12 hours, a white ZnO-SnO_2_ powder was produced.

Characterization 

Following synthesis, characterization of the NPs was carried out at the White Lab-Material Research Centre, Saveetha Dental College and Hospital, Chennai. The morphological study of the NPs was performed by scanning electron microscopy (SEM) imaging. The structural study was performed by X-ray diffraction (XRD) analysis and the chemical studies were performed by energy dispersive X-ray spectroscopy (EDAX) and FT-IR spectroscopy.

Morphological study: SEM imaging

High-resolution SEM, in conjunction with an EDAX diffractometer (Model: JEOL, JSM IT-800), was employed to analyze the surface characteristics of the NPs. Samples of ZnO-SnO_2_ NPs were mounted on carbon-taped aluminum stubs, gold-coated using a sputter coater, and seen under an SEM. 

Structural study: XRD

**Figure 1:**
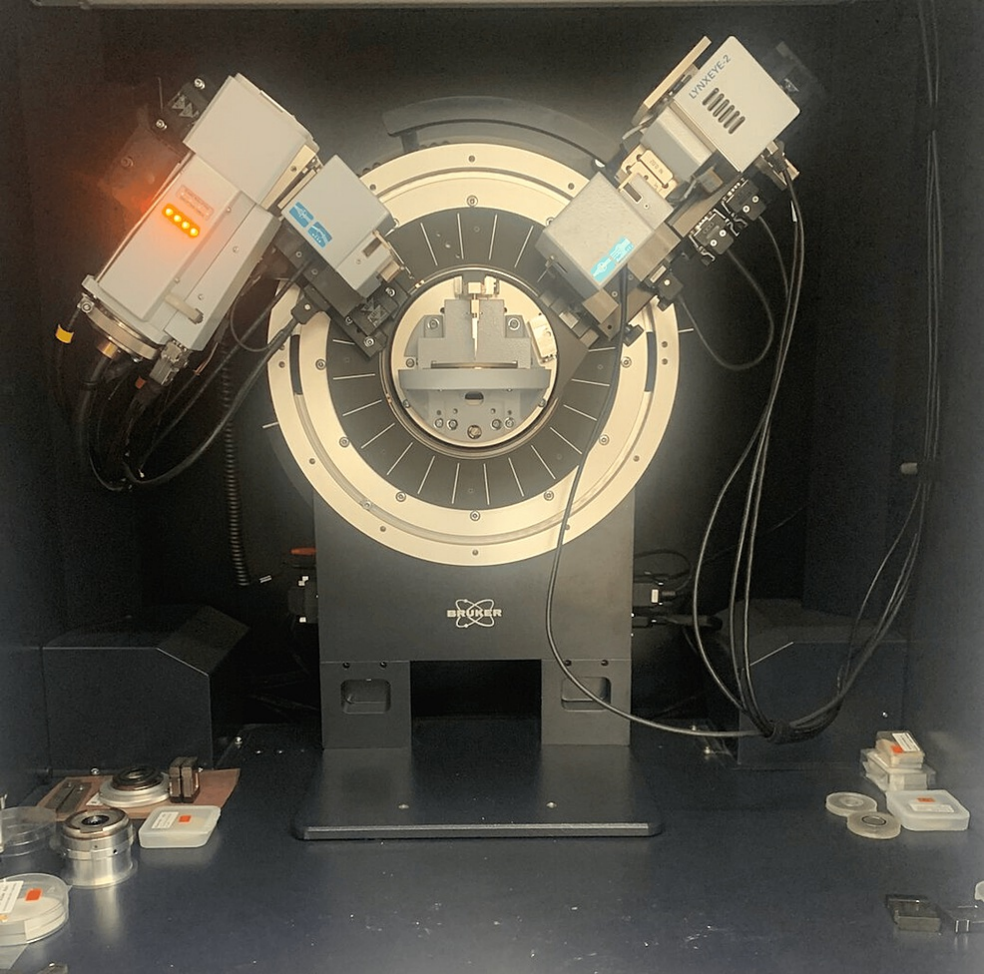
Bruker D8 Advance

This technique provides detailed information about the crystallographic structure of the NPs based on the interference of a crystalline sample and monochromatic X-rays. In XRD analysis, the X-rays generated are collimated and directed to a sample of the NPs, and the incident rays interact with the sample to produce a diffracted ray that is detected, processed, and counted. Understanding the crystallinity and crystal structure of the NPs was carried out by the powder XRD analysis in the 2ϴ range of 20 - 80° (Model: Bruker D8 Advance, Bruker Corporation, USA) (Figure 1). The plot of the diffraction pattern displays the intensity of scattered diffracted rays at different angles of the material.

Chemical studies: EDAX and FT-IR spectroscopy

**Figure 2:**
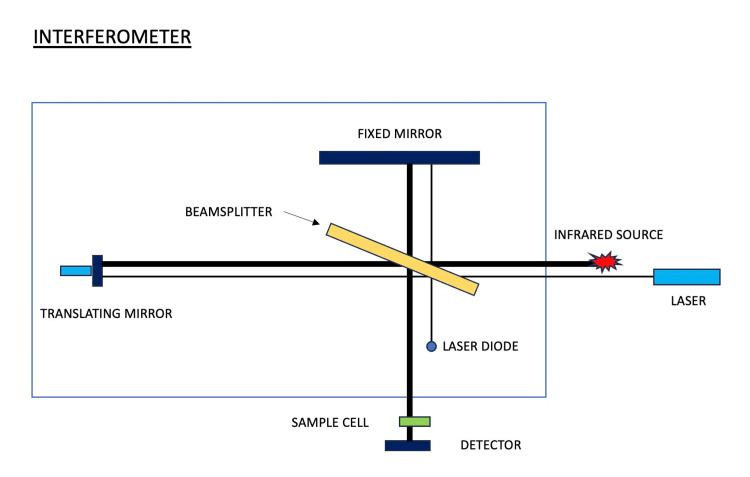
INTERFEROMETER

The elemental composition of the ZnO-SnO_2_ NPs was determined for EDX analysis. In this technique, the NPs are analyzed by activation using an EDX spectrophotometer. The bonding and functionality of the NPs were studied using Fourier transform infrared spectroscopy. FT-IR spectrometer comprises a test chamber, source, amplifying device, detector, computer, and an analog-to-digital converter. The interferometer allows radiation from the sources to travel to the detector. An analog-to-digital converter and amplifying device form a digital signal that is produced by an amplified and converted signal, followed by the transfer of the signal to the computer where the Fourier transform is carried out. A portion of the infrared radiation, which has a wavelength of roughly 4000-500cm^-1^ (Bruker-ALPHA 2, Bruker Corporation, USA), is transmitted through the sample while the remaining energy passes through partially (Figure 2). The sample transforms the radiation absorbed into rotational or vibrational energy. The final signal produced at the detector has a spectrum that typically ranges from 4000 to 500 cm^-1^, and it represents the molecular signature of the samples. The films' infrared (IR) spectra were taken using a Fourier transform infrared spectrometer. 

Antibacterial test

An agar diffusion test was performed to evaluate the antibacterial properties of the NPs. The antibacterial properties of two concentrations of the NPs, ZnO + SnO_2_ (30 μg/ml), ZnO + SnO2 (15 μg/ml), and Control (Antibiotics) were assessed. Antibiotic erythromycin was used against gram-negative and penicillin was used against gram-positive bacteria. All the Petri plates were filled with nutrient agar (25 ml each) and were left to harden. 50 μl of S. aureus, S. mutans, and E. coli cell suspension was pipetted onto an agar plate. Three indentations were created on the agar using the gel puncture method with adequate spacing between each well and the plates were placed in the incubator for 16-18 hours at 37°C. The inhibited bacterial zone was assessed encircling all the wells and the diameter was measured and documented in millimeters.

## Results

Morphological evaluation of the synthesized NPs using SEM

**Figure 3:**
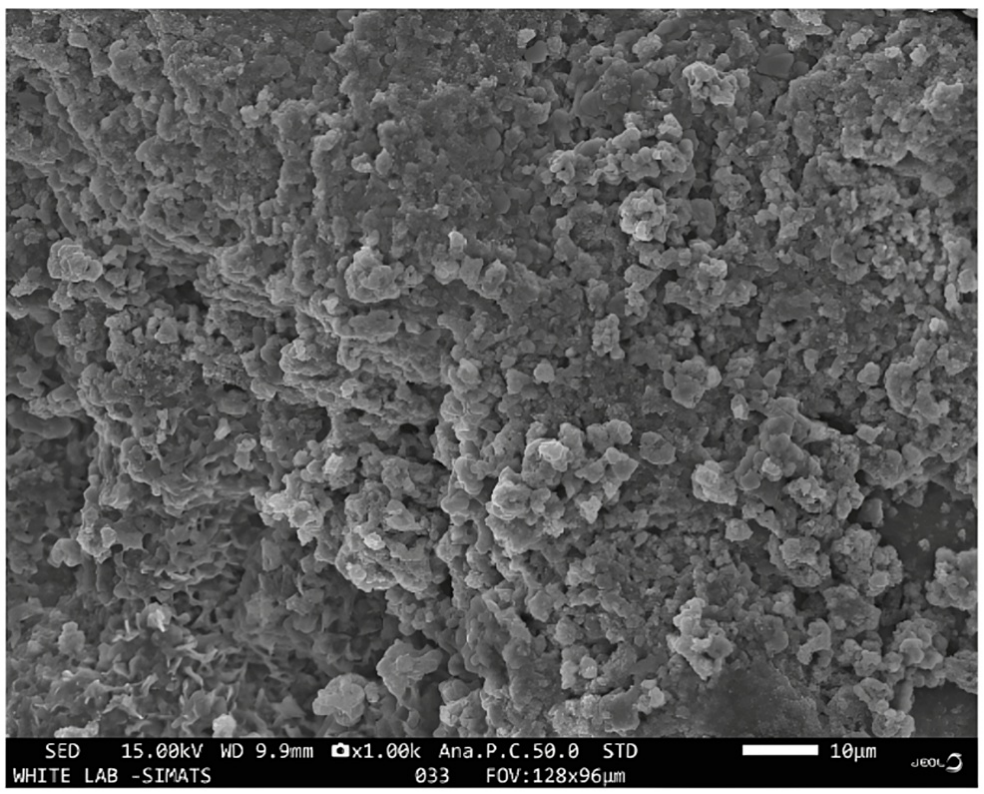
Scanning electron microscopy image of the prepared ZnO-SnO nanoparticles

Field emission microscopy (SEM) was used to determine the morphology of the synthesized SnO_2_:ZnO NPs. Figure 3 shows that NPs were big, asymmetrical particles. The existence of both SnO_2_ and ZnO NPs in the nanocomposites is further supported by the observation of some irregular flower-shaped NPs. The surface structure is characterized by compact nanosized particles. These particles tend to aggregate, posing a challenge to accurately measuring the size of individual nanoparticles. Moreover, some particles fuse, forming clusters with a notably high degree of agglomeration.

Chemical studies: EDAX spectroscopy 

**Figure 4:**
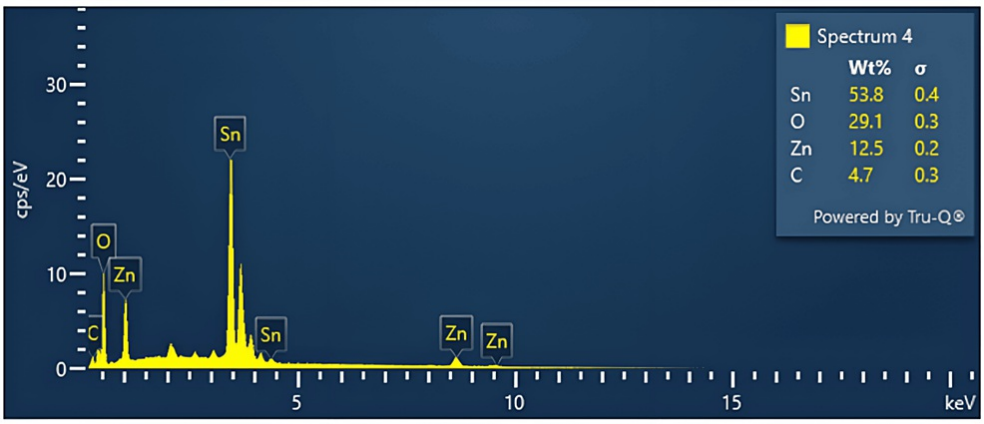
Graph showing the chemical composition of ZnO-SnO2 nanoparticles using EDAX spectroscopy X-axis: keV (energy unit); Y-axis: cps/eV (counts per second per electron volt); Sigma: error in weight percentage

Figure 4 shows the graph of the EDAX spectrum showing the composition of the constituents which were Sn- 53.8 Wt%, C-4.7%, Zn- 12.5 Wt%, and O- 29.1 Wt%. The weight percent and atomic percent of the constituent elements match the weight percent of the synthesized gel, further demonstrating the purity of the synthesized nanocomposite. The weight percentage (Wt%) of each element was calculated based on the intensity of the peak height for each element. The distribution of the highest peak intensity corresponds to the highest observed weight percentage of the respective element in the nanoparticle. Specifically, it was observed that the peak intensity of the Sn element surpassed that of other elements. The analysis of the data revealed that no additional contaminants were generated during the synthesis process.

FTIR spectroscopy

**Figure 5:**
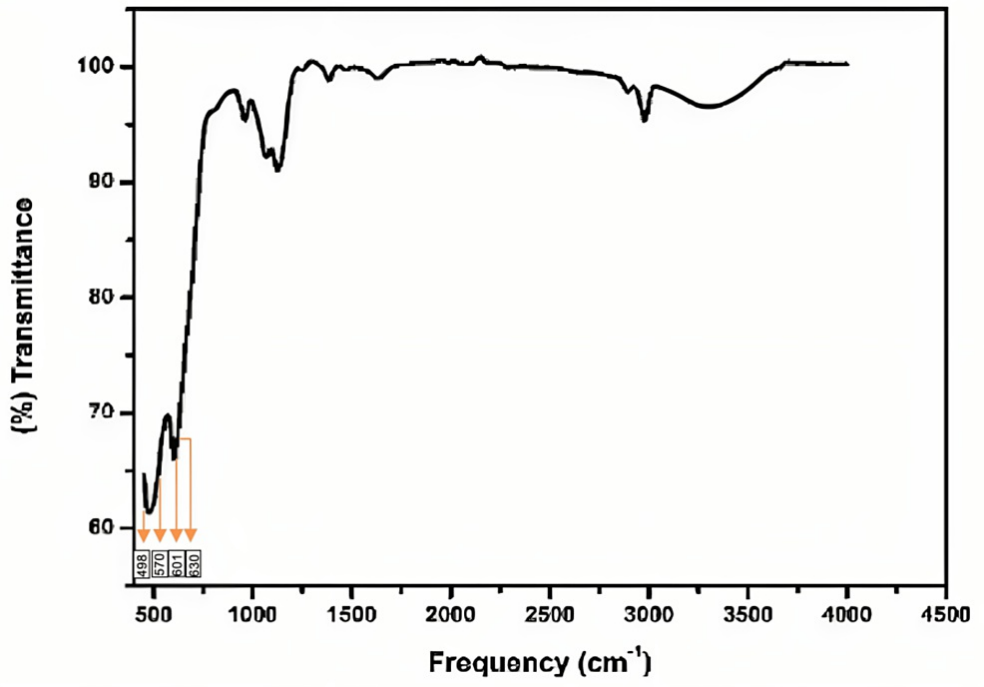
Graph showing Fourier transform infrared spectroscopy results of the prepared ZnO-SnO nanoparticles

Figure 5 shows the functional groups found in pure SnO_2_:ZnO nanocomposites as determined by FTIR analysis. In the peaks at region 570 cm^-1^, 601 cm^-1,^ and 630 cm^-1^ [20], the typical peak of tetragonal SnO_2_ NPs was seen. Bands of vibration emerging between 1600 and 3473 cm^-1^ in the sample were the result of the -OH molecules' bending and stretching vibrations [21]. The bands that occur in the peak region between 1400 and 2373 cm^-1^ were used to identify the absorption of CO_2_/organic moieties from the atmosphere [22,23]. A new peak corresponding to Zn-O stretching was seen in nanocomposites at 498 cm^-1^. This suggests that SnO_2_ was successfully combined and formed the nanocomposites of SnO_2_:ZnO. Although the presence of both ZnO and SnO_2_ nanoparticles had an impact on the overall functional structure of the ZnO:SnO_2_ nanocomposite, a notable difference in peak shapes was observed between the two.

Structural study: XRD analysis

**Figure 6:**
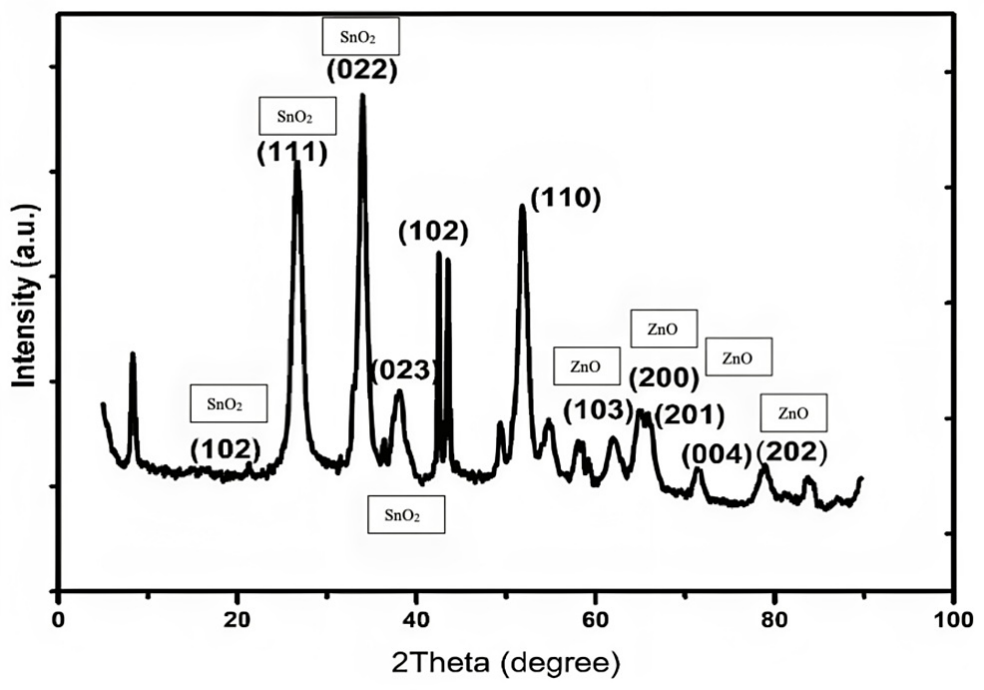
XRD pattern of the prepared ZnO-SnO2 nanoparticles

XRD pattern examination provides a brief insight into the surface shape and crystalline nature of the synthesized nanocomposites. The appearance of sharp peaks in the XRD pattern of NPs indicated a high degree of crystallinity. The observed plane result indicated that the polycrystalline nature was observed for SnO_2_: ZnO nanocomposite. New peaks corresponding to SnO2 NPs crystalline planes (102), (111), (022), and (023) occur in the XRD patterns of SnO_2_:ZnO nanocomposites at angles of 21.81°, 25.37°, 32.91°, and 35.96° respectively. According to JCPDS card no. 78-1063, these peaks represent the orthorhombic structure of SnO_2_. The peak positions at 62.81°, 66.37°, 67.91°, 72.96°, and 76.92° are related to the diffraction planes of (103), (200), (201), (004) and (202) respectively. The observed planes indicated that the hexagonal structure of ZnO was based on the JCPDS card no. 780-0075. Figure 6 provides the XRD pattern of the prepared ZnO-SnO_2_ NPs. The XRD patterns of all samples reveal peak broadening, indicating the nano-crystalline nature of the materials. Micro-strain in the XRD pattern is attributed to deficiencies within the crystalline lattice, such as vacancies, stacking faults, and interstitials. Notably, a change in crystalline phases and/or crystal structure was observed in SnO_2_, suggesting the formation and incorporation of ZnO into the SnO_2_ NPs.

Antibacterial properties

**Figure 7:**
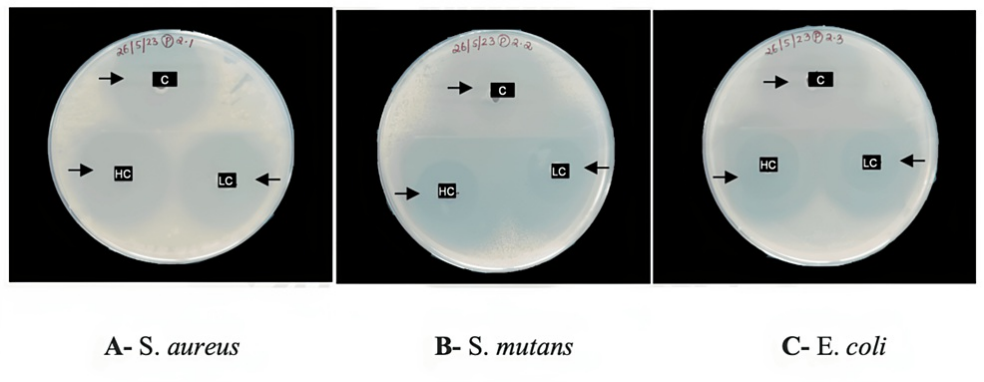
Zone of inhibitions were observed around the high- and low-concentration nanoparticles and the control (antibiotics) against S. aureus, S. mutans, and E. coli. C-control, HC-High concentration, LC-Low concentration

The antibacterial activity of the synthesized NPs against the bacterial cultures of S. aureus, S. mutans, and E. coli at high (30 μg/ml) and low (15 μg/ml) concentrations is shown in Figure 7. Agar plates A, B, and C showed the antibacterial activity of the NPs against S. *aureus*, S. *mutans,* and E. *coli* respectively. A zone of inhibition is noted around the NPs. Table 1 provides the zone of inhibition values of high and low concentrations of NPs against the tested bacteria. It was observed that the chemically synthesized ZnO- SnO_2_ NPs showed a similar zone of inhibition as compared to the control at both concentrations. 

**Table 1:** Zone of inhibition values of high and low concentrations of nanoparticles against the tested bacteria in mm

Bacteria	Control	Low concentration (15 μg/ml)	High concentration (30 μg/ml)
S. aureus	20 mm	24 mm	26 mm
S. mutans	24 mm	26 mm	28 mm
E. coli	25 mm	30 mm	31 mm

## Discussion

In the present study, ZnO-SnO_2_ NPs were synthesized followed by evaluation of their morphological, structural properties, and antibacterial properties at two different concentrations. The ZnO-SnO_2_ NPs synthesized by the sol-gel technique displayed an irregular shape in their morphological examination. Chemical analysis confirmed the composition of the elements as follows: Sn- 53.8 Wt%, Zn- 12.5 Wt%, and O- 29.1 Wt%. Additionally, it confirmed the presence of a chemical bond between ZnO and SnO_2_ NPs. The structural assessment revealed that the ZnO and SnO_2_ NPs had hexagonal and orthorhombic structures respectively. Furthermore, when assessing the zone of inhibition of these NPs at varying concentrations against S. aureus, S. mutans, and E. coli, it was observed that their inhibitory effects were comparable to the control group. 

The structural study performed by XRD analysis observed a value of 0.2868 nm was found to be in accordance with the (022) plane of SnO_2_, the interplanar spacing value of 0.3383 nm calculated from the lattice image shown in the XRD pattern is well-matched with the values observed in XRD (0.3337 nm), which corresponds to the (110) plane of ZnO [24]. The produced ZnO:SnO_2_ nanocomposites' polycrystalline characteristic is determined by the XRD pattern. These results were well in agreement with a previously published study by Zarei et al. which confirmed the purity and high crystallinity of the ZnO-SnO2 thin films [25]. 

It has been noted that combining two or more potentially lethal NPs to produce nanocomposites results in an increase in antibacterial activity. The suggested mechanism beyond the antimicrobial activity of NPs is based on the generation of reactive oxygen species (ROS) that have the capacity to upset protein activities, disrupt the secondary membrane, and damage DNA [26]. Numerous investigations have investigated the fact that ROS can still be formed even in the absence of light. ZnO-SnO_2_ NPs have the capacity to generate a high number of reactive oxygen species. Therefore, the generation of reactive oxygen species is the only factor responsible for the increased activity of SnO_2_-ZnO nanocomposites [27]. Zn2+ interacts with the cell wall, leading to the depletion of the cell membrane. This causes the intracellular fluids to leak out, resulting in the loss of viability of the cells. Tin interacts with the physiological pathways in cells, which disturbs the regular process and ultimately results in the death of the bacterial cells. Pandey et al. found an improvement in the anti-bacterial activity of ZnO−Ag_2_O/Ag, ZnO−CuO, and ZnO−SnO2 nanocomposites made using the solvochemical approach compared to their single metal oxide equivalents [28]. It was noted that the antibacterial activity was higher at a lower concentration and increased after 3 hours. Evstropiev et al. studied the zone of inhibition of ZnO−SnO2 nanocomposites and concluded that the incorporation of SnO_2_ with ZnO NPs increased the antibacterial activity against gram-positive and gram-negative bacteria [29]. It was concluded that the addition of SnO_2_ NPs led to a decrease in the crystal size of ZnO crystals and increased the surface area of the nanocomposites, which increased their antibacterial properties. In the present study, the zone of inhibition at both high and low concentrations of the NPs was noted to be comparable to the control group. The ZnO-SnO_2_ NPs can be further studied for cytotoxicity and surface morphology when coated on orthodontic brackets and archwires to reduce friction and can be incorporated into restorative adhesives to enhance the bond strength. 

Limitations

Various factors such as pH, temperature, pressure, and light can influence the synthesis process, potentially leading to variations in the properties of the NPs. Higher concentrations of the NPs may pose toxicity concerns, impacting their safety in various applications hence future study is required. The assessment of antibacterial activity in vitro might not cover the full spectrum of bacterial strains.

## Conclusions

ZnO-SnO_2_ NPs were effectively synthesized by the sol-gel technique, which is a cost-effective method. The synthesized NPs were characterized by SEM, EDAX, XRD, and FT-IR spectroscopy, signifying their structural, morphological, and chemical properties. The NPs showed a good antimicrobial effect against the tested bacteria, suggesting that the NPs could be used for inhibition of bacterial growth.
